# Understanding the nexus between gender-based violence and mental health among Anglophone internally displaced women in Littoral Cameroon through an intersectional socioecological lens

**DOI:** 10.21203/rs.3.rs-9118674/v1

**Published:** 2026-04-02

**Authors:** Patrice Gérard Ngangue, Douglas Mbang Massom, Daniele Sandra Yopa, Philippes Mbevo Fendoung, Jeanne Claire Dissak Delon, Theodore Tameh, Fanny Nadia Dissak Delon

**Affiliations:** Laval University; Epicentre; Ministry of Public Health; National Advanced School of Public Works; Institut Universitaire des Grandes Ecoles des Tropiques; University of Bamenda; University of Bamenda

**Keywords:** Internally Displaced Persons, Gender-Based Violence, mental health, intersectionality, Cameroon, qualitative research, socio-ecological model

## Abstract

**Background:**

Internally displaced women (IDW) affected by the “Anglophone crisis” in Cameroon navigate a landscape of overlapping vulnerabilities, including displacement-related poverty, gender-based violence (GBV), and systemic linguistic minoritization. While the psychological impact of war is well-documented, the mental health consequences of intersecting urban stressors in host settings like Douala remain underresearched.

**Objective:**

To explore how displacement, GBV exposure, and Anglophone identity intersect to shape the mental health experiences and resilience strategies of IDW in Douala, Cameroon.

**Methods:**

This qualitative study utilized semi-structured interviews with 33 Anglophone IDW (Median age: 34 years); residing in the Littoral Region for a median duration of 5 years). Participants were recruited via purposive and snowball sampling from various urban neighborhoods (e.g., Bonabéri, Mbanga). Data were analyzed through reflexive thematic analysis using NVivo 12, guided by an intersectional socioecological framework.

**Results:**

Four major themes emerged: (1) “Swallowed Silence,” where emotional repression functions as a strategic social protection against stigma; (2) Embodied Trauma and Cognitive Paralysis, manifesting as somatic distress and dissociation; (3) Chronic Survival Anxiety, driven by the “scarcity mindset” of economic precarity and ethno-linguistic discrimination; and (4) Adaptive Resilience, anchored in informal peer networks, spirituality, and cognitive restructuring.

**Conclusion:**

Mental health among Anglophone IDW is a dynamic process shaped more by ongoing structural “daily stressors” than by past events alone. Interventions must transcend clinical models to integrate GBV prevention, livelihood protection, and culturally safe MHPSS services delivered in English and Pidgin.

## Background

Cameroon is currently navigating a complex landscape of multifaceted and protracted humanitarian crises [1]. Since late 2016, the socio-political conflict in the North-West and South-West Regions—often referred to as the “Anglophone Crisis”—has escalated into a significant armed confrontation, precipitating a massive internal displacement of populations [2]. Current humanitarian estimates indicate that over one million individuals are internally displaced within the national territory [1]. This displacement is not a static event but a fluid and precarious process of “pendular movements” and temporary returns, reflecting a persistent state of ontological insecurity. For those fleeing to Francophone metropolises like Douala and surrounding cities, the transition from rural or semi-urban conflict zones to a dense, competitive urban environment often marks the beginning of a second wave of invisible violence: structural marginalization [3].

In contexts of armed conflict, the domestic and public spheres merge into a continuum of risk for women and girls [4]. Displaced women are disproportionately exposed to heightened protection risks, where Gender-Based Violence (GBV)—including intimate partner violence (IPV), sexual exploitation, and systemic harassment—is not merely a byproduct of war but a tool of social destabilization [5, 6]. In the urban “anonymity” of Douala, these risks are amplified by acute economic precarity, substandard housing in informal settlements, and the erosion of traditional community safety nets [7]. The clinical literature consistently demonstrates that the psychological toll of GBV extends far beyond immediate physical harm [8]. Survivors frequently exhibit a complex constellation of symptoms, including clinical depression, Post-Traumatic Stress Disorder (PTSD), and debilitating anxiety [9, 10]. However, in the specific context of displaced Cameroonian Anglophones, these symptoms are often intertwined with chronic somatization—where psychological distress is expressed through physical pain—and long-term functional impairments that hinder their ability to rebuild their lives [11].

Relocation to Douala, while offering relative physical safety from active combat, introduces a “complex layering” of urban stressors. For Anglophone women, the struggle for livelihoods and informal employment is exacerbated by linguistic and ethno-identity discrimination [12]. In a predominantly Francophone socio-economic ecosystem, the “Anglophone identity” can become a marker of “otherness,” influencing everything from the price of rent to the probability of being hired or harassed by local authorities. This linguistic barrier is not merely a communication issue; it is a barrier to “therapeutic citizenship.” It shapes how these women perceive their own safety and belonging, often leading to a sense of “hostile hospitality” where they are physically present but socially excluded. This marginalization is rooted in the historical and political grievances that fueled the crisis, now re-enacted in the daily micro-aggressions of urban life [13].

To capture this multidimensional distress, mental health must be reconceptualized beyond clinical pathology [14]. The Inter-Agency Standing Committee (IASC) Guidelines (2021 update) underscore that well-being is contingent upon multi-sectoral synergy—integrating protection and economic dignity [15]. Consequently, a Socio-Ecological Model is indispensable for mapping distress from the individual through the microsystem to the macrosystemic forces of linguistic policy [16]. This is sharpened by an Intersectional Framework [17], which posits that being “a woman,” “displaced,” and “Anglophone” produces a unique configuration of marginalization. This shifts the focus from resilience as a trait to resilience as a negotiated survival strategy [18]. Despite the scale of displacement, a void persists regarding the subjective realities of Anglophone IDWs in Douala. While reports quantify “needs,” they bypass how women navigate the triad of distress, stigma, and survival [19]. Specifically, there is a lack of inquiry into how linguistic identity dictates the use of silence—as both a protective shield and a psychological prison [20]. To address this gap, this study drew on semi-structured interviews with 33 Anglophone IDW residing in the Littoral Region of Cameroon, specifically in various neighborhoods of Douala (such as Bonabéri) and surrounding cities (such as Mbanga). By focusing on women primarily active in the informal sector, this research explores the lived realities of those navigating the fringes of the urban economy. The objective was to understand how mental distress and resilience were coconstructed by intersecting social identities and the structural conditions of their displacement.

## Methods

### Theoretical framework

This study was informed by a dual, complementary framework that situates mental health as a socially embedded process rather than a purely individual outcome. By integrating an intersectional lens with a socio-ecological model, the analysis was able to bridge the gap between psychological distress and the structural realities of displacement in Cameroon.

The intersectional framework [17, 21] guided the examination of how overlapping social positions— specifically gender, displacement status, and Anglophone identity—interact to shape lived experiences of vulnerability, stigma, and resilience. Rather than treating these dimensions as isolated variables, intersectionality facilitated an analysis of how structural inequalities and power relations are experienced simultaneously. This was essential for understanding how the “Anglophone identity” in Francophone towns like Douala and surrounding towns/villages, functions as a unique vector of marginalization that compounds gender-based risks. Simultaneously, the socio-ecological model [16] informed the interpretation of findings across multiple levels of influence. This model allowed the study to map the fluid pathways of distress from the individual level (e.g., embodied trauma and cognitive paralysis) through the microsystem (interpersonal dynamics and peer support), up to the exosystem and macrosystemic forces (structural poverty, linguistic discrimination, and national policy). Together, this dual framework provided a robust theoretical basis for analyzing mental health as a product of continuous negotiation between war-related trauma, urban insecurity, and the search for social legitimacy. It shifted the focus from resilience as an innate personality trait to resilience as a negotiated survival strategy exercised within a deeply constrained social field

### Study design

A qualitative interpretive study design was adopted, drawing on an experiential and constructivist approach to understanding mental health in contexts of displacement [22].

This design was chosen because the study sought to explore how internally displaced women interpret and give meaning to experiences of distress and coping within specific socio-political and economic contexts. Quantitative approaches would have been insufficient to capture the layered, contextdependent, and socially mediated nature of these experiences. Qualitative inquiry, by contrast, allows for an in-depth exploration of perceptions, narratives, and relational processes, which is particularly relevant when studying sensitive topics such as violence, stigma, and psychological suffering.

The study therefore aimed not to measure prevalence but to generate a contextualized understanding of how distress is experienced, articulated, and managed in everyday life.

### Study setting

The study was conducted in the Littoral Region, mainly in its city capital Douala, which is Cameroon’s main economic hub and a major urban destination for internally displaced persons fleeing conflict in the North-West and South-West regions. As a result of our snowball sampling, some of the participants that were recommended lived in small towns and villages around Douala, namely Souza and Mbonjo. Participants resided in various neighbourhoods characterized by heterogeneous living conditions, including informal housing arrangements, overcrowded dwellings, and precarious rental situations. These urban/semi-urban conditions provided a relevant context for examining how displacement intersects with livelihood insecurity and social integration challenges.

### Participants and inclusion criteria

Participants were eligible if they (1) were women aged 18 years or older, (2) identified as Anglophone internally displaced persons, (3) had relocated to Douala or around Douala due to the conflict in the North-West and South-West Regions, and (4) provided informed consent.

Participants were excluded if their emotional state did not allow safe participation at the time of recruitment.

### Recruitment procedures

Participants were recruited through purposive sampling, complemented by snowball sampling. Initial contacts were established through community networks and local non-governmental associations of displaced persons. Snowball recruitment allowed access to individuals who might otherwise be reluctant to engage with formal institutions or research processes. This approach facilitated trust-building and ensured diversity of experiences.

### Data collection

Data were collected between February and March 2025 through individual semi-structured interviews. The interviews explored participants’ displacement histories, exposure to insecurity or violence, economic survival strategies, social relationships, emotional responses, and coping mechanisms. Interviews were conducted in English or Cameroonian Pidgin English, depending on participants’ preferences, in locations ensuring privacy and safety. Each interview lasted approximately 30 minutes and was audio-recorded with consent. Field notes were taken to document contextual observations and researcher reflections.

### Data collection tools

A semi-structured interview guide was developed based on existing literature on GBV, displacement, and mental health, as well as the socio-ecological framework. The guide included open-ended prompts designed to encourage narrative accounts while ensuring coverage of key domains such as stigma, safety, economic challenges, and support networks.

### Data analysis

All interviews were transcribed verbatim and managed using NVivo 12 software.

Data were analyzed using reflexive thematic analysis, following the six-phase approach described by Braun and Clarke: (1) familiarization with the data through repeated reading of transcripts, (2) generation of initial codes capturing meaningful features of the data, (3) searching for patterns and potential themes across transcripts, (4) reviewing themes to ensure internal coherence and distinction, (5) defining and naming themes, and (6) producing an interpretive narrative linking themes to the theoretical framework [23, 24]. The analysis combined inductive coding, allowing themes to emerge from participants’ narratives, with deductive interpretation guided by the intersectional and socio-ecological lenses. This process enabled the identification of multi-level determinants of distress and resilience. Throughout the analysis, attention was paid to preserving participants’ meanings while situating their accounts within broader structural contexts.

### Ethical considerations

Participation was voluntary and based on informed consent. The consent was taken directly from the participants for adults (>=21years); for minors (<21years) we took the assent from the participant as well as informed parental consent from the parent or an adult guardian. Participants were informed about the purpose of the study, their right to withdraw at any time, and the measures taken to ensure confidentiality. Given the sensitivity of the subject matter, interviews were conducted in private settings, and pseudonyms were used in transcripts and reporting. Participants who experienced emotional distress during interviews were provided with information on available community and psychosocial support resources.

In accordance with Cameroonian procedures, the study received ethical approval from the Regional Human Health Research Ethics Committee for the Littoral and the Administrative Research Authorization from the Regional Delegation of Public Health for the Littoral.

## Results

### Participant characteristics

A total of 33 Anglophone internally displaced women (IDW) participated in this study, providing a diverse cross-section of the displaced population in Douala. The participants ranged in age from 16 to 53 years, with a median [IQR] age of 34 [30, 41] years. representing various life stages—from young women pursuing their education to older women responsible for multi-generational households. On average, participants had been residing in the Littoral Region for 5 years, indicating a transition from emergency displacement to a state of protracted urban settlement.

The socio-economic profile of the participants was characterized by high levels of resilience within the informal economy. Most were economically active in the informal sector, reflecting the dominant livelihood strategies available to displaced populations in urban settings. The majority were engaged in precarious yet vital livelihood activities, including: Petty trade: Selling foodstuffs (garri, vegetables or candies) in their neighbourhoods; domestic and service work: Working as cleaners, laundry workers, or in small-scale catering. Home-based production: Such as hairdressing or preparing street food. Educational backgrounds were diverse, spanning from those with primary education to university graduates.

Housing conditions were a significant stressor, with participants living in overcrowded rental units in neighborhoods such as Bonabéri, often sharing limited space with other displaced families. Furthermore, most participants disclosed a history of conflict-related insecurity or GBV, including experiences of harassment during flight, domestic tension linked to economic stress, and linguistic-based microaggressions in the urban space. These socio-economic realities constitute the “ecological niche” in which their mental health experiences are embedded.

### Overview of thematic findings

The thematic analysis identified four major themes illustrating how participants’ mental health was shaped by a continuous negotiation between war-related trauma, urban insecurity, and everyday survival pressures. Across narratives, mental health emerged not as a fixed condition but as an ongoing process in which internally displaced women navigated between the need for silence to preserve social stability and the persistent expression of trauma through bodily reactions and survival anxiety.

#### Theme 1 — “Swallowed Silence”: Emotional Repression as Strategic Social Protection

The most pervasive theme identified across the narratives was the deliberate and systematic internalization of suffering. Participants frequently employed the metaphor of “swallowing” their problems, describing it as the only viable response to a landscape defined by limited institutional protection, economic dependency, and pervasive social stigma. In this context, silence was not a passive state of wordlessness, but an active, strategic performance of “normalcy.”

#### 1.1 Silence as a Shield for Social Legitimacy

For many women, silence functioned as a mechanism to preserve their fragile social legitimacy within the host community and their own families. In a context where Anglophone IDPs are already marginalized, any disclosure of “dishonor” (such as sexual harassment or marital conflict) risked total reputational collapse.

“I would break my image (…) I would not have a voice among people (…) when I try to say something, they will say ‘you, that woman who slept with someone’s husband’ (…) I will not have a voice to sit anywhere.”

— Participant PDI3

“I cannot ask him because I know that if I ask him if it is not beating (…) I was forced to accept everything.”

— ParticipantPDI11

This quote illustrates that silence is a negotiated trade-off: the woman exchanges her psychological relief (speaking out) for the preservation of her “social voice” and right to belong to the community.

#### 1.2 The Economic Rationale for Silence

The choice to remain silent was also rooted in a pragmatic assessment of the judicial and social systems. Participants viewed formal reporting as a “luxury” they could not afford, linking silence directly to their economic precarity.

“For me, I always swallow it (laughs). That is the only thing. Because if you want to go and make a report, how much do you have?”

— Participant PDI6.

“We reported him. The thing is just that they asked for money from my mother, money for fine, my mother didn’t have money to give. So they just abandoned the case.”

Participant PDI14

Here, the “laugh” noted in the interview reflects what researchers call “dark humor” or “subversive irony,” a way to mask the bitterness of knowing that justice in the urban host setting is often transactional.

#### 1.3 Gendered Blame and “Secondary Victimization”

Fear of blame—a form of secondary victimization—was particularly acute among survivors of sexual harassment or attempted assault. The participants highlighted a socio-cultural environment where the burden of proof and morality is placed solely on the woman.

“Because when such a thing happens, they do not blame the man (…) they blame the woman. They will say: ‘why didn’t you refuse?’”

— Participant PDI3

“I could not tell his wife (…) she would have thought it was something we had already done.”

— Participant PDI3

These narratives demonstrate how patriarchal norms and marital expectations intersect with the vulnerability of displacement. Silence becomes a “prison of safety”: it protects the woman from immediate social ostracization but traps the trauma within the body, potentially leading to the “embodied distress”.

#### Theme 2 — Embodied Trauma and Cognitive Paralysis: The Somatization of Insecurity

For the participants, trauma was not a distant memory of conflict, but a deeply embodied and everpresent reality. The narratives reveal a transition from psychological distress to physiological manifestations, where the body continues to react to a perceived environment of threat, even in the relative safety of the host city.

#### 2.1 The Somatic Signature of Fear

Participants described intense, involuntary bodily responses triggered by the presence of perceived aggressors or the recollection of violent events. These reactions—palpitations, trembling, and acute autonomic arousal—illustrate how trauma is “stored” in the body.

“When he came, my heart started beating fast (…) it was afternoon and nobody was in the house (…) I felt you came just for that thing [rape].”

— Participant PDI3

“When he insults me, it hurts my heart very much and it worries me a lot.”.

— Participant PDI1

This “fast-beating heart” is a somatic signature of the “freeze-fight-flight” response, exacerbated by the isolation of the domestic space (“nobody was in the house”), which remains a site of potential revictimization in Douala.

#### 2.2 Cognitive Paralysis and Dissociative States

Beyond physical symptoms, trauma manifested as a form of cognitive paralysis. Participants described a total shutdown of agency during threatening encounters—a state of being “lost” where language and action become impossible.

“When you face such a circumstance, you shut down (…) you are lost (…) you don’t know how to talk (…) I just sit as if I have nothing to do, afraid the man wants to destroy my life.”

— Participant PDI3

“I was scared to talk to him… I was just scared! Because maybe, if I want to talk, maybe he will not answer, or he will just shout at me.”

— Participant PDI4

“I was forced to behave like a dump [dumb], someone who cannot talk because I understood that even in his mistakes… I didn’t have time and energy to draw his attention.”

— Participant PDI11

This “shutting down” aligns with clinical definitions of dissociation or the “tonic immobility” response often seen in survivors of prolonged violence. In an intersectional sense, this paralysis is not just psychological; it is reinforced by the woman’s lack of social and legal power to “talk back” to her aggressor.

#### 2.3 Hypervigilance and the “Precarity of Space”

The trauma was chronically reinforced by the instability of urban living conditions. In Douala’s overcrowded and insecure housing, the boundary between the “safe” private sphere and the “threatening” public sphere is blurred.

“I was afraid he might come to the house when my husband is not there… because one day I was inside and he came.”

— Participant PDI3

“At Douala things were not easy… food and a place to sleep are not easy… if you don’t have money, they can chase you anytime.”

— Participant PDI6

These narratives highlight a state of structural hypervigilance. The fear of being “chased anytime” due to economic precarity mimics the initial trauma of forced displacement from the North-West/South-West. Thus, the urban environment does not heal the trauma; it provides a new stage for its repetition, where the threat of eviction or hunger functions as a secondary trauma.

#### Theme 3 — Chronic Survival Anxiety: The Interplay of Economic Precarity and Linguistic Discrimination

Economic insecurity was not merely a backdrop to the participants’ lives; it emerged as the primary driver of psychological distress, often perceived as more destabilizing and “noisier” than past episodes of physical violence. This “survival anxiety” is a form of chronic stress where the immediate need for food, shelter, and education consumes the mental bandwidth necessary for trauma recovery.

#### 3.1 The “Parental Guilt” and Educational Precarity

For many participants, the most acute psychological burden was the inability to fulfill their roles as providers, particularly regarding their children’s education. In Anglophone culture, education is often seen as the ultimate tool for social mobility; thus, its interruption is experienced as a profound failure and a source of deep anxiety.

“For me, the highest risk is seeing children not go to school; that disturbs me greatly… Sometimes they are sent home for school fees… if I had something permanent, it would not affect them like this.”

— Participant PDI6

« Sometimes, they drive [them from school]. The time they stay long without me giving money… Even now, while talking, the children are at home. »

— Participant PDI1

The lack of “something permanent” (stable employment) creates a cycle of instability where the mother’s mental health is tethered to the daily fluctuations of the informal market.

#### 3.2 Linguistic Minoritization and Employment Barriers

The intersectional nature of their distress is most visible in the labor market. Participants reported that their Anglophone identity acted as a “stigma” that limited their access to even the most menial jobs, transforming their language into a marker of political suspicion or perceived incompetence.

“When I said North-West, she said ‘no, no, no’ (…) she cannot accept someone from that section [region].”

— Participant PDI6

“They insult IDPs… they say ‘those are Bamenda people who ran from war’… they say we are not intelligent.”

— Participant PDI3

These micro-aggressions and “ethno-linguistic insults” (e.g., the pejorative use of “Bamenda”) reinforce a sense of alienation. The women are not just “displaced”; they are “othered” in their own country, which exacerbates their anxiety by creating a sense of “hostile hospitality” in the urban space.

#### 3.3 The Fragility of the Support System: Widowhood and Abandonment

The burden of survival was often carried alone, due to the death or absence of male partners—a common outcome of the conflict and the stresses of displacement. This forced autonomy in a competitive city like Douala creates an overwhelming psychological weight.

“We came to Douala and after some time my husband died, leaving three children (…) how do you become stable? It is not easy.”

— Participant PDI3

« Sometimes he leaves immediately he beats me and he will [be] about two days or more out of the house before he returns. »

— Participant PDI1X

The death of a spouse is not only a personal grief but a structural catastrophe that removes the “microsystemic” buffer against the “macrosystemic” pressures of poverty. The resulting state is one of “chronic precariousness,” where the woman must navigate a patriarchal urban economy without any formal or informal safety net.

#### Theme 4 — Resilience Mechanisms: Cognitive Adaptation and Informal Pillars of Support

Despite the density of their psychological and structural burdens, the participants demonstrated a profound capacity for resilience. In the context of this study, resilience was not defined by the absence of distress, but by the strategic mobilization of internal and collective resources to maintain psychological equilibrium and hope.

#### 4.1 “Strategic Cognitive Avoidance”: Protecting the Mind

One of the most common adaptive strategies was a form of deliberate cognitive avoidance. Far from being a passive denial, this was a conscious decision to “seal off” distressing memories and future anxieties to prevent total emotional collapse and maintain the ability to function as breadwinners.

“I decided for now I will not put anything in my mind… because when I think about it, it worries me and could make me do something I don’t want.”

— Participant PDI3

“Working hard… Keeping myself busy I get to forget my struggles.”

— Participant PDI1.

This “intentional forgetting” serves as a protective barrier. In a precarious urban environment where the participant cannot afford to be incapacitated by grief, cognitive avoidance becomes a tool for survival, allowing them to remain focused on the “immediate present.”

#### 4.2 Peer Networks: Informal Therapeutic Ecologies

In the absence of formal, culturally safe mental health services, displaced women reconstructed their own informal psychosocial spaces. These networks, often formed with other women in similar situations, functioned as “therapeutic ecologies” where experiences were validated and practical survival strategies shared.

“I have two friends, we sit and talk about life… how to move forward, how to help the children succeed.”

— Participant PDI3

« Sometimes it is good to share, it is not everybody that you have to share with, but at least some key people. »

— Participant PDI2

These gatherings represent a mesosystemic level of resilience. By focusing their dialogue on “moving forward” and the success of their children, these women transform their shared trauma into a collective project of future-building. This informal solidarity acts as a buffer against the linguistic and social isolation they face in the wider city.

#### 4.3 Spirituality as an Ontological Anchor

Spiritual faith emerged as the most consistent pillar of resilience, providing an explanatory framework for their suffering and a source of perceived protection. In a world where institutional and social protection is absent, “God’s grace” becomes the ultimate safety net.

“It is just by God’s grace (…) God is a God who does not change (…) I work with that.”

— Participant PDI6

“I pray that God should never let me fall into that kind of situation [again].”

— Participant PDI3

For these women, spirituality provides ontological security—the sense that despite the chaos of displacement and the “hostile hospitality” of Douala, there is an unchanging, higher order. This faithbased coping allows them to externalize their burdens and find the moral strength to endure daily microaggressions.

## Discussion

This study explored the mental health experiences of Anglophone internally displaced women living in Douala, Cameroon, highlighting how displacement, gender-based violence, and economic precarity intersect to shape distress and resilience.

This study demonstrates that the mental health of Anglophone IDWs in Douala is not a static byproduct of past conflict-related trauma, but a continuously re-actualized state driven by the “slow violence” of urban marginalization [25]. While clinical models often prioritize the “event” of violence, our findings suggest that for displaced women, the post-migration environment is equally pathogenic [26]. This resonates with the work of Miller and Rasmussen [19, 27], who argue that “daily stressors” (poverty, lack of services) are often stronger predictors of psychological distress than direct war exposure. In Douala, the intersection of gender and linguistic minoritization creates a syndemic effect, where structural inequities and psychological trauma mutually reinforce each other [28].

A primary contribution of this study is the conceptualization of “Strategic Silence.” Our findings move beyond the view of silence as a mere symptom of emotional repression. Instead, we argue that “swallowing” distress is a form of negotiated agency. By choosing silence, participants actively manage their “social capital” in a host environment where they lack institutional safety nets [29]. As Essue et al. (2025) argue, silence in urban displacement is often a response to ‘structural gaslighting’—where the survivor recognizes that reporting violence in a linguistically hostile environment like Douala offers no path to economic safety, only further fragmentation [6]. Furthermore, the ‘Political Muting’ described by Tanyi (2023) explains why Anglophone survivors may perceive silence as a necessary defense against being profiled as political suspects by the host community [30]. In Douala, this silence is also a tool for “linguistic passing”—minimizing one’s Anglophone identity to navigate a Francophone urban hierarchy. This signals a critical gap in protection systems: when the cost of seeking justice is the loss of social legitimacy, silence becomes the only rational, albeit painful, survival strategy [31].

The centrality of survival anxiety illustrates how economic precarity functions as a cognitive tax. Participants’ focus on “school fees” and “house rent” reflects what is described as the “scarcity mindset,” which consumes the mental bandwidth necessary for trauma processing [32]. Our findings contrast with some Western-centric trauma models that focus primarily on intra-psychic recovery [33]. In the context of Douala, mental health is inextricably linked to livelihood security. This mirrors findings from Abramowitz (2014) in post-conflict Liberia, where “disturbances in the mind” were frequently articulated through the language of economic abandonment [34]. The “moral distress” of not being able to educate their children highlights how parental roles are a key site of psychological vulnerability for IDW women [35].

Finally, this study challenges the neoliberal view of resilience as an individual trait. By highlighting peer networks and spirituality, our results align with Panter-Brick’s (2014) ecological framework, which views resilience as the “capacity to navigate toward resources [18]. The use of “Strategic Cognitive Avoidance” is particularly noteworthy. While traditional clinical psychology might label this as “maladaptive,” in the context of persistent urban uncertainty, it serves as a necessary functional coping mechanism [36]. It allows women to remain economically active in the informal sector despite their trauma [37]. Furthermore, the role of spirituality as an ontological anchor mirrors studies by Pargament (2011) and Tanyi (2002) in the African context, where religious coping provides a sense of continuity and meaning in the face of profound displacement [38, 39].

A significant finding of this study is the deeply embodied nature of trauma. The physiological symptoms reported—heart palpitations, “shutting down,” and chronic hypervigilance—indicate that trauma is not a memory of the past but an active, somatic presence. This aligns with Bessel van der Kolk’s (2014) seminal work on how the body “keeps the score” of violence [34]. In contexts like Douala, where psychological services are scarce, distress is often communicated through somatization. As Kirmayer (2001) suggests, in many non-Western cultures, bodily symptoms are not just “signs” of distress but the primary language of suffering itself [40].

The persistence of fear within the domestic sphere further suggests that trauma is reinforced by ontological insecurity [41]. Our findings support a shift in humanitarian mental health: moving from a focus on PTSD as a fixed “post-event” disorder toward a dynamic, ecological understanding of distress produced by a lack of safety in the present [19].

Our data reinforces the growing consensus that economic insecurity is a primary, rather than secondary, driver of psychological distress in displaced populations. For the participants, the “mental tax” of school fees and housing instability was often more debilitating than the memory of conflict. This echoes Miller and Rasmussen (2010), who argue that “daily stressors” are frequently more predictive of poor mental health than direct war exposure [27].

In the urban informal markets of Douala, this anxiety is compounded by linguistic discrimination. The “othering” of Anglophone women through ethno-linguistic insults (e.g., “Bamenda”) creates a barrier to “therapeutic citizenship”—the feeling that one has a right to health and safety in the city. Thus, mental health in this context must be analyzed as an outcome of structural violence, where political exclusion and economic marginalization are “written” onto the psychological well-being of the displaced [42].

Despite these pervasive stressors, the study highlights that resilience is a socially embedded process rather than an individual trait. The use of peer networks as “informal psychosocial spaces” confirms the importance of social capital in navigating displacement. These networks function as what Panter-Brick (2014) calls “ecologies of resilience,” providing both emotional validation and practical survival strategies [18].

Similarly, spirituality acted as a central ontological anchor. This resonates with research across subSaharan Africa showing that religious belief provides a framework of meaning that clinical interventions often lack. In this sense, resilience among Anglophone IDWs is not the “absence of suffering” but a form of negotiated agency—the capacity to maintain hope, social connection, and dignity within a structural environment that actively militates against their well-being [38, 39].

Taken together, the findings support a socio-ecological reconceptualization of mental health in displacement contexts. Distress in Douala is not a static clinical state but a fluid interaction between individual trauma, interpersonal power dynamics, community exclusion, and structural insecurity [43]. Consequently, this study reinforces the IASC (2007) guidelines, advocating for mental health and psychosocial support (MHPSS) that is integrated into broader protection, livelihood, and social inclusion frameworks [44].

Given that economic precarity is a primary driver of anxiety, livelihood support (e.g., micro-grants or vocational training) should be recognized as a preventive mental health intervention [45]. Reducing “survival anxiety” is a prerequisite for any successful trauma-focused therapy. There is an urgent need for MHPSS services delivered in English and Pidgin English in Douala. Such services must move beyond Western talk-therapy to include somatic-based approaches and peer-led support groups, which bypass the “strategic silence” observed in formal settings [46].

Addressing the linguistic stigma and ethno-identity discrimination is not merely a political necessity but a public health requirement. Urban integration policies must actively combat the “othering” of Anglophone IDPs to foster a sense of belonging and psychological safety.

### Limitations and Directions for Future Research

This study has limitations inherent to its qualitative and contextual nature. Since most of our participants lived in a specific urban hub (Douala), the findings are not transferable to rural IDPs or those in other linguistic regions.

Furthermore, while the snowball sampling and recruitment through specialized local associations facilitated trust, a degree of social desirability bias may have influenced disclosures regarding sensitive topics like GBV or political grievances. The cross-sectional design offers a “snapshot” of lived experiences but does not capture the longitudinal trajectories of recovery or the shifting nature of “shame” and “resilience” over years of displacement. Future research should employ longitudinal mixedmethod designs to evaluate how specific economic and protection interventions directly correlate with mental health outcomes over time.

The strengths of this study lie in its focus on the under-documented intersections of Anglophone identity, gender, and displacement in Cameroon. By grounding the analysis in rich, qualitative narratives, we have moved beyond the “quantification of needs” to understand the quality of suffering and the agency of resilience.

## Conclusion

This study demonstrates that the mental health of Anglophone internally displaced women (IDW) in Douala is not a static clinical outcome of past conflict, but a dynamic, socially-produced state shaped by the ongoing interaction of economic insecurity, ethno-linguistic stigma, and structural marginalization. The narratives reveal that psychological distress is deeply embedded in the “slow violence” of everyday urban survival, while resilience is actively negotiated through informal social ties, strategic cognitive adaptation, and spiritual resources.

These findings underscore a critical mandate for humanitarian and public health actors: to move beyond narrowly individualistic, clinical approaches toward integrated socio-ecological interventions. Effective responses must synchronize gender-based violence (GBV) prevention with livelihood protection and culturally-grounded psychosocial support that leverages existing community ecologies. Ultimately, addressing the structural barriers to social inclusion—specifically linguistic discrimination and economic precarity—is a prerequisite for the sustainable mental well-being of displaced populations in Cameroon. By foregrounding the lived realities of IDWs, this study contributes a nuanced, intersectional perspective to the global discourse on urban displacement, emphasizing that mental health is inseparable from the pursuit of social justice and economic dignity.

## Figures and Tables

**Table 1:** Socio Demographic Characteristics of the Participants (N = 33)

Socio Demographic Characteristics	n (%)	Values
Age (years)		
min; max		16; 53
median [IQR]		34 [30, 41]
Length of stay in the Littoral Region (years)		
min; max		1; 12
median [IQR]		5 [3, 7]
Occupation		
Unemployed	6 (18)	
Student	1 (3)	
Farmer	8 (24)	
Informal Trader	8 (24)	
House help	1 (3)	
Hairdresser	3 (9)	
Factory worker	1 (3)	
Teacher	2 (6)	
Nanny/Nursery attendant	2 (6)	
Cash collector	1 (3)	
Marital Status		
Single	8 (24)	
Married	6 (18)	
Widow	7 (21)	
Divorced	3 (9)	
Partnered single	7 (21)	
Partnered divorced	1 (3)	
Partnered widow	1 (3)	
Level of Education		
None	1 (3)	
Primary	13 (39)	
Secondary	16 (48)	
Higher Education	3 (9)	
Town of residence		
Douala	19 (58)	
Mbanga*	4 (12)	
Souza**	4 (12)	
Mbonjo***	6 (18)	

*: semi-urban area located 71km west of Douala;

**: rural area located 38.7km west of Douala;

***: rural area located 39.5km west of Douala.

## Data Availability

The data that support the findings of this study are not openly available due to reasons of sensitivity and are available from the corresponding author upon reasonable request.
